# Affective Neuroscience: The Suitability of a Web App to Monitor Affective States at Work

**DOI:** 10.3389/fpsyg.2021.592143

**Published:** 2021-06-04

**Authors:** Paola Manfredi, Elena Massardi

**Affiliations:** ^1^Department of Clinical and Experimental Sciences, University of Brescia, Brescia, Italy; ^2^University Research Center Integrated Models for Prevention and Protection in Environmental and Occupational Health (MISTRAL), University of Brescia, Brescia, Italy; ^3^Self Employed, Clinical Psychotherapist and Organizational Consultant, OPL, and NPSA Association, Brescia, Italy. Registered at the professional psychological association, Lombardia, Italy

**Keywords:** basic affective systems, work, work wellness, web-app, Panksepp, clinical psychology, management, neuro-psychoanalysis

## Abstract

This work describes in detail the use of a new tool, a web-app, based on the conceptual framework of affective neuroscience, in particular on Panksepp’s 7 basic emotional systems. Affective neuroscience has been used effectively in many areas, but there have been very few applications in the workplace, due to the lack of a smart implementation tool. The novelty of this work does not lie in the new information, but in a new “clinical” approach. There is a theoretical framework that allows data to be interpreted rather than simply described. Furthermore, the knowledge of working realities through the web app is specific and longitudinal. Finally, emotions are detected in *hic et nunc*, so the role of reflexive-cognitive mediation and recall bias are minor. This “more situated” knowledge can then guide specific leadership strategies. This paper presents the results of the tool’s application in a company in Northern Italy. The findings of our project, which recorded basic affective states and the functioning of several working teams, are detailed herein. The project’s 488 web-app records are summarized in this report, alongside our examination of related mood tags. Through this project, our analysis has enabled to determine affective neuroscience profiles of the teams analyzed, allowing the researchers to identify areas of possible interventions. The data appear very encouraging.

## Introduction

In the past decades, scientific literature highlighted that the affective dimension has a key role in determining workplace well-being and productivity. A clear indicator that highlights the recognition of the importance of affective dimensions is, for example, the integration of the traditional Karasek model (Karasek, 1979) with the social support component (Job demand – control – social support) (Karasek and Theorell, 1990).

Progressive attention to more psychological and affective aspects is also found in relation with topics of more recent interest, such as sustainability. De Jonge and Peeters state that “Sustainable work systems are systems where human, job, and social resources are: regenerated and renewed through the process of work while still maintaining productivity” (De Jonge and Peeters, 2019 p. 2).

Initially, analyzing the concept of sustainability, it has been emphasized the physical dimension rather than the social environment dimension, encompassing only more recently psychological aspects of well-being at work, including understanding how employee health, well-being, and performance are related to long-term productivity and vitality (Pfeffer, 2010). Therefore, it becomes relevant stimulating employee’s vitality to generate positive and enduring socio-ecological outcomes (De Jonge and Peeters, 2019). There are different definitions of vitality, which can be ordered according to progressive levels of complexity. Vitality can refer to: power of enduring, energy and strength, physical and/or mental vigor, capacity to live and develop, positive feeling of having energy available to the self, and finally to a specific psychological experience of possessing enthusiasm and spirit. The latter meaning refers to a state of physical and psychological health, which contemplates self-actualization, agency, personal well-being, and growth (De Jonge and Peeters, 2019).

The complexity of the affects and emotions considered on one side gives a measure of how deep the level of the research is, but on the other side opens up new problems, such as finding appropriate indicators and survey instruments for evaluations.

The construction and use of adequate tools must be consistent with a methodological choice. Research on employee emotions includes very different methodologies and frameworks. For example, the analysis of an individual dimension may be preferred to that of a group. In the case of the individual dimension, affects can be considered as an individual property, delimited by a person’s physical or psychic skin. In this perspective, the accent is placed on the peculiar characteristics of the individual, on personal abilities and on the representations of oneself and of the environment (Schusterschitz et al., 2018). The interrelation between the characteristics of the person and the working environment determines a promotion vs. deterioration of health. In the case of the group dimension, attention will be paid to the dynamics that emerge in the work environment, in horizontal and vertical relationships.

Another difference in study perspectives is to consider affects and emotions inherent to the organization itself, as in the systemic-psychodynamic approach (Trist et al., 1997). The systemic-psychodynamic approach highlights that the emotional experience appears to be a property of a human context, or of a relational context. The work environment is no longer only the place where emotions of individuals find expression, it is no longer a context among others, but it is, as a work organization, “an eliciting object of emotion” (Armstrong, 2019, p. 22).

In brief, the transition is from emotional life *in* organizations to emotional life *of* organizations. In this perspective, emotions become a key to access the functioning of the organization. Therefore, they mainly represent an indicator that can allow understanding the organization, the tasks and the challenges of that particular working reality. From this point of view, it could be said that just as the emotions of the individual are involved in psychic well-being, so work emotions can be linked to the well-being of the workplace. Hence, as we investigate a patient’s emotions for diagnostic purposes, we might likewise detect the emotions of the working organism for an assessment.

In this study, we are interested in exploring the expression of emotions in a work environment, understanding emotions as characterizing the work organism, and finding out how to measure the dynamics of this aspect in everyday experience. Our perspective finds its roots in affective neuroscience (Panksepp and Biven, 2012; Panksepp et al., 2017; Solms, 2019) and in Tavistock’s systemic-psychodynamic approach (Jaques, 1971; Hirschhorn and Gilmore, 1992; Hutton et al., 1994; Armstrong, 2005, 2019).

The hypothesis being tested is that using affective neuroscience when working with organizations can help to detect emotional undertow and improve management and team performance. We are also interested in analyzing how emotions evolve in teams and how they can be elicited by work-related or non-work-related events.

The aim of this paper is to assess the possibility of designing a smart tool to record affective dynamics, and to test its effectiveness in a sample of workers. We do not present a definitive version of this instrument, but we rather propose a feasibility study to assess whether the prototype can:

(a) Be easily used by workers,
(b) Allow to detect significant elements in teams’ emotional dynamics, and so if it is possible to find differences in the emotional functioning among different teams,
(c) Help finding depictions in which workers can recognize themselves, and how these depictions can be used to help make the organization more agile, more responsive and provide foundations for dialogue between managers and teams.

## The Tool

### Theoretical Premises

In order to investigate emotions as expressions of the group and constitutive of a team (see section “Introduction”), the first question we asked ourselves is which significant emotions, indicative of the team functioning, are mainly expressed at work; secondly, how to design a tool that can detect these emotions and also monitor the flow of emotions’ dynamic changes over time.

We are well aware that there is a wide range of investigated emotions in literature. Our choice was to refer to the current studies of affective neuroscience, and to the paradigms of institutional analysis and socio-analysis rooted in systemic-psychodynamic theories. Both systemic-psychodynamic theory and affective neuroscience have highlighted the central role of our emotions in determining how we respond to the outside world.

The psychoanalytical tradition underlines that in organizations we can meet primitive affects and defenses in action (Bion, 1948; Bleger, 1966; Jaques, 1970; Anzieu, 1975; Kaes, 1976; Kaes et al., 1988). As members of a cooperative system, we are emotionally and cognitively tied together, and feelings aren’t just the inevitable emotional residue of human work relationships: “they are data, valuable clues to the dynamics of boundary relationships. In this respect, feelings are an aid to thinking and to managing; they are a real part of real work” (Hirschhorn and Gilmore, 1992, p. 115). We can therefore assume that, because human beings are emotional animals and “the work is a human invention, serving human purposes and dependent on human beings to function” (Armstrong, 2019, p. 11) primary affects are activated in this environment.

Recent neuroscientific studies (Panksepp and Biven, 2012; Davis and Montag, 2019) have shown that we humans share at least seven basic core emotional systems with other mammals; these emotions are rooted in the deepest part of our brain, and emerge from deep ancient brain networks, which control our instinctual emotional life. These emotions are extraordinary tools in supporting the survival of the individual. The “over-ruling” of our deep emotions is done by other parts of the brain because “All aspects of mental life can be influenced by our primary-process feelings, and the overall affective spectrum of the lower Midbrain is foundational for higher mental health issues” (Panksepp and Biven, 2012, p. XX).

In the context of work organizations, the basic emotional systems could be compared to background music, fundamental in constituting the organizational climate. The awareness of these emotions, and of their particular composition in each specific working reality, can reinforce the emotional, relational and problem-solving skills of managers, team leaders and employees of an organization.

Many and diversified emotions may be experienced at work, but the complex affects are based on the combination of basic emotional systems.

Of course, “a great deal of cortical-cognitive competence is permitted by neo-cortical regions, but it has long been known that upper brain-mind regions cannot operate effectively without the genetically dictated subcortical emotional-affective, motivation and consciousness sustaining systems” (Montag and Panksepp, 2017, p. 3).

Panksepp and Biven (2012) identified seven core systems of emotions in the brain as: SEEKING^1^, RAGE, FEAR, LUST, CARE, PANIC/GRIEF, and PLAY.

Montag et al. (2021) describe the different systems as follows: “SEEKING, LUST, CARE, and PLAY represent the positive primary emotional systems, whereas ANGER, FEAR, and SADNESS can be found on the negative emotional side”.

To illustrate this, herein below an overview the evolutionary functions of the positive primary emotional systems is shown:

• SEEKING system channels the energy of the mammalian brain to seek life resources, from food or a partner to safety. This basic motivation-emotional system provides us with energy in everyday life to fulfill our goals and it participates in all our emotional systems.
• LUST system is of importance for reproductive reasons for sustaining our species.
• CARE system elicits emotional urges to care for our offspring, so they can grow into adults and have offspring themselves.
• PLAY is of tremendous importance for social bonding and for shaping skills in the area of social competencies and motor functions as well as possibly contributing to neocortical regulation of emotions.

As for the negative primary emotional systems:

• RAGE/ANGER system protects life resources including our life or the lives of loved ones and may also be triggered by physical restraint.
• FEAR system keeps us away from bodily harm and “physical pain.” “It helps us cope with immediate danger by triggering a freezing or flight response that can be specifically influenced by the defensive distance between prey and predator” (…).
• PANIC/GRIEF system is the neural circuitry underlying the sadness. From an evolutionary point of view our sapiens species naturally lives in groups and we feel “psychological pain” “when we are separated from our loved ones and when we find ourselves socially isolated and alone” (Montag et al., 2021, pp 160-161).

From these 7 “primary” emotion systems, secondary emotions emerge (empathy, trust, pride, blame, guilt, shame, etc.). Finally, at the cortex level, we have a tertiary system: distancing system, containment, mentalization, names of feelings, mindfulness (Panksepp and Biven, 2012). Adult humans have a chance to experience a wide range of emotions and affects. Life experiences, relationships, learning, cognitive development, reflective functions (in other words secondary and tertiary processes) expand and make emotional life more complex. However, emotional systems also form the basis for more complex emotions: they are, so to speak, an elementary language, but essential and common to all living beings.

Panksepp (2006) proposed a connection between basic emotional systems and the expression of emotions, as shown in the following Table 1.

**TABLE 1:** Postulated relationships (first and second column) between basic emotional systems and common emotional processes (Panksepp, 2006).

**Basic emotional systems**	**Emergent emotions**	**Emotions at work**
SEEKING (+)	Interest Craving	Engaged
SEEKING (−)	Frustration	Frustrated, Bored
RAGE (+ and −)	Anger Irritability Contempt Hatred	Angry
FEAR (−)	Simple anxiety Worry Psychic trauma	Stressed
PANIC (−)	Separation distress Sadness Guilt/shame Shyness Embarrassment	Sad, Isolated
PLAY (+)	Joy and glee Happy playfulness	Playful, Happy
PASSION (+) (LUST)	Erotic feelings Jealousy	Excited
CARE (+)	Nurturance Love Attraction	Valued, Confident

*Plus, and minus signs after each item indicate major types of affective valence that each system could presumably generate. The last column lists emotions at work used in the web-app.*

### Tool’s Characteristics

Our view is that the challenge with answering the question of how to evaluate the emotions’ impact on an organization currently begins with a process for collecting, analyzing and reporting data about the emotions triggered during the working day. For this reason, we wanted to test the use of a web app tool to record the emotional states of workers on a daily basis. In working teams, emotional states were collected to build and compare the emotional profiles that, possibly, characterize them differently.

We have chosen to privilege the use of a simple, connected, and immediate tool such as the web app, as opposed to the administration of questionnaires, because we believe that this registration method requires the subject to make an “immediate” and limited choice of the emotion experienced in the “here and now,” while also limiting bias recall. In this way, a judgment or evaluation of the prevailing and overall state of the individual is not required, and the focus is on the present emotional state.

We wanted to translate affective neuroscience findings into a simple tool (web app) to measure in real time and daily the emotional activation of individuals and teams and provide insights for users and leaders over time and throughout different areas/teams. At the same time, the measuring tool we offer also gives readings of the organization response to real time events (such as new strategical and organizational choices) becoming an insightful lab for natural experimentation.

Our methodology is based on the following steps:

1. Measuring in real time the most prevalent systems in an organization by recording emotions of each employee, emotions being chosen from a set presented by the app.
2. The recording gives the trends (timelines).
3. Through tagging some causes the user can explain why some systems are currently engaged.

**FIGURE 1:**
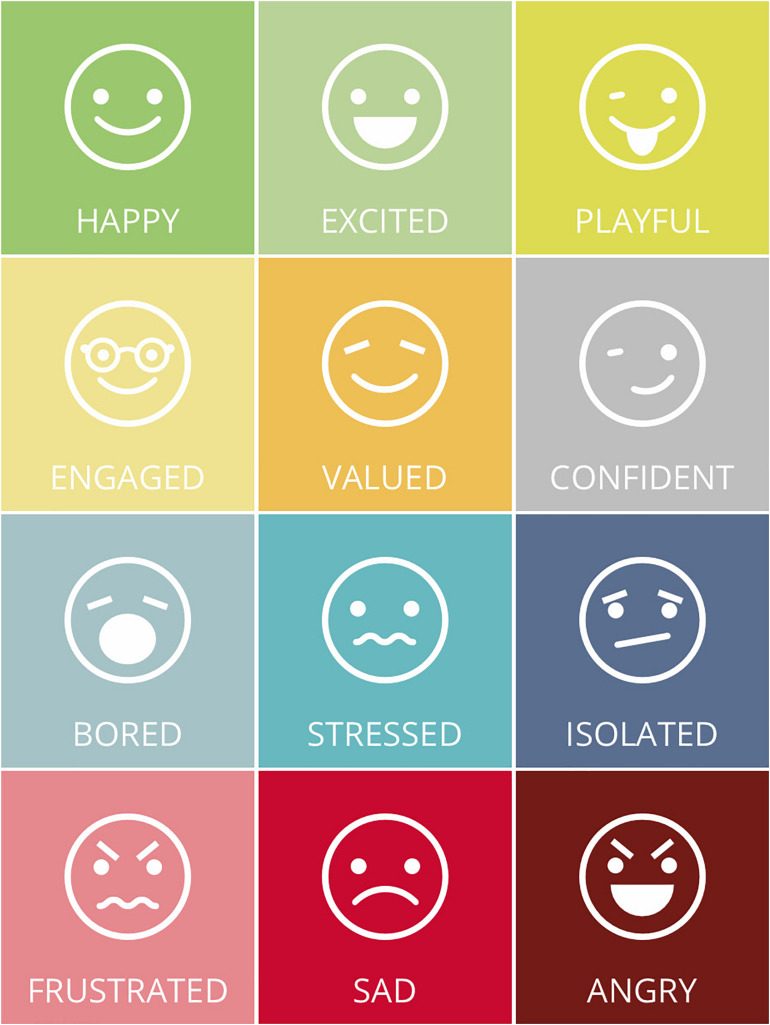
Set of emotions presented by the app.

The first screen of the app displays the 12 emotions (Figure 1) based on Panksepp’s postulated relationships between basic emotional systems and common emotional processes that we deemed to be more relevant to the working field.

The emotions are displayed as emoticons to facilitate the recognition thereof, and to give a sense of easiness and lightness. The worker can choose one or more emotions that best describe what he/she is feeling or what he experienced on a specific day. The user interacts with the app daily, possibly at the beginning and at the end of the day.

After selecting the emotion, users are asked to explain, using a free text box and/or predefined tags, the reason why they feel that way (Table 2).

**TABLE 2:** Example of emotions and connected tags.

**Emotions**	**TAG**
Happy	Good outcome
Excited	It will be a productive day
Angry	Forced to go on holiday
Angry	Women prejudice
Confident	Work to do

The app provides the opportunity to record own feelings, get a feedback (metrics), reflect through recording own thoughts. The emotion choice is stored in a timeline and associated to the user profile (individual recordings remain anonymous). The individual will have the opportunity to look back at his/her own thoughts and past results.

**FIGURE 2:**
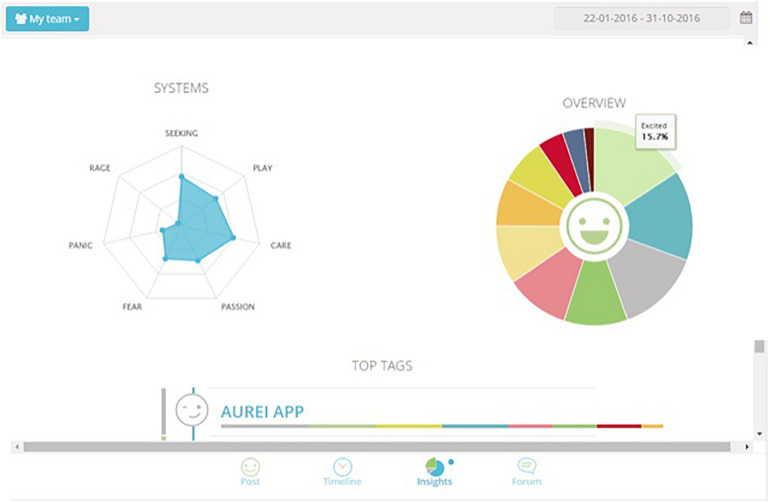
Team collected data. The cumulative percentages of choice of emotions made by a team are represented in the pie chart (Different colors match to the different emotions as they are displayed in Figure 1) while the web graph shows the correspondence between chosen emotions and emotional systems.

Feedback can be viewed by each user individually but also at a team level, based on specific selected time periods. The data are processed and aggregated in a way that provides insights on the emotional profile of users and on the emotional profiles of the team he/she belongs to. In this way, the level of awareness on the group’s well-being is increased and it is possible to elicit discussion, sharing and support between colleagues and managers regarding the management of the team’s “emotional capital.” Figure 2 shows how the data are displayed, that is with a spider web graph showing which emotional systems have been most activated in the selected recording interval, followed by the representation of the percentages of emotions in a pie chart.

The tool’s timeline function also permits the individual user to question how coherent their general representation is, compared to the chosen daily emotions. For example, the user who is questioned on an evaluation of their work through a questionnaire, could define the work as “not very satisfying and demanding.” But observing the timeline of the recorded emotions, they could notice that the affective states are mainly positive. The next question could therefore be the evaluation of the causes of this sense of “heaviness”: other areas of one’s life could contaminate the working environment (i.e., getting to work already fatigued by other problems can bear upon the evaluation of the working environment). App users may become aware of the fact that they tend to attribute their suffering to work, rather than questioning themselves about their personal life, or discovering that some expectations and desires may have changed.

In summary, research undertaken via questionnaire implies a general evaluation over a long period, while the punctual, “real-time” survey bypasses, as far as possible, some superstructures that we may summarize as attributable to character variables. We believe that in this way, the focus is more upon what the subject feels, rather than on what they think about what they have felt.

Although the tool focuses on the basic affective states, these are integrated by further analysis, because in addition to the choice of the emotional state, the user is asked to tag the event that is believed to have triggered the emotion. This provides important elements for developing a deeper understanding of the origins of emotional states. For example, if an apparently trivial event activates strong emotions, such as panic, we could assume that in that team there is a (chronic) lack of CARE system activation. The expression of the tags also allows us to compare how the same event is experienced by different subjects or in different teams. Finally, the tool also makes it possible to evaluate to what extent events originating in different contexts (e.g., family) continue to influence workplace emotions, in positive or negative terms.

## Pilot Study

### Materials and Methods

#### Methodology

A pilot study was conducted to assess the applicability of the tool, and its ability to “profile” work situations.

For this purpose, a large company in northern Italy has been identified, sufficiently healthy from a financial performance perspective, and without high risks of assessment of work-related stress.

This research is part of a larger research protocol titled “The water challenge: smart models, tools, and methods for assessing environmental suitability and effects of green technologies on human health.” Therefore, the Company did not ask for a psychological intervention, but rather it asked for an availability to take part to a research mainly focused on water depuration technical aspects. In compliance with Italian regulations, the Company had already drafted the documentation for evaluation of work-associated stress, and employees had already been involved in filling of questionnaires and in focus groups. Our collaboration was possible thanks to our proposal of a different evaluation tool, a small influence on working times and our commitment to provide relevant aggregated data to the managers involved, and by e-mail to every participant to the research.

The actual recording time, excluding contacts with people in charge, trade unionists and workers, was 4 weeks, from November to December.

The research, in compliance with the Helsinki protocol, was authorized by senior management, and presented to the workers concerned in meetings which took place in all of the branches of the company. The workers concerned filled in the informed consent form and were subsequently sent via e-mail a summary of the study, some instructions on the use of the tool, and a private password to access their personal section on the web app portal.

#### Sample

A total of four meetings have been organized, one in the headquarters in Verona and the others in three company branches in the province, to present the research and explain how the app works. Unfortunately, attendance by workers was very poor. Some workers lamented a late communication of the event from the company and therefore the difficulties for some colleagues to be present. Some others inferred that the low number of participants could derive from a failure from the company to return the outcomes of past projects.

The company sent the workers an e-mail containing a brief summary of the research and an invitation to contact the researchers if interested. In case of acceptance, further details and the informed consent form were transmitted. After receiving the signed informed consent form, the researchers provided each worker with private username and password for accessing the app. The majority of the sample consisted of people who took part to one of the informative meetings.

The sample was made up of 41 people, with a total of 488 registrations in 4 weeks of surveys.

The sample was divided in several teams, in compliance with the company organization chart.

### Results

The data were aggregated to provide feedback on the activation of emotional systems on three levels: for the participating individual, for the teams included in the trial, and also for the entire company.

**FIGURE 3:**
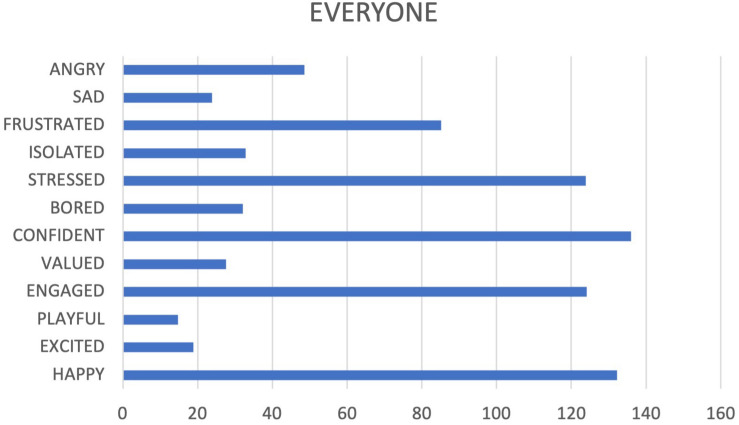
Cumulative frequencies of all participant’s data input.

The input data were analyzed alongside the corresponding descriptive indicators and the cumulative frequencies of the participant’s data input (Figure 3). We represented these data in graphs. We then carried out a qualitative analysis, which integrated the emotional choices with the detected tags.

**FIGURE 4:**
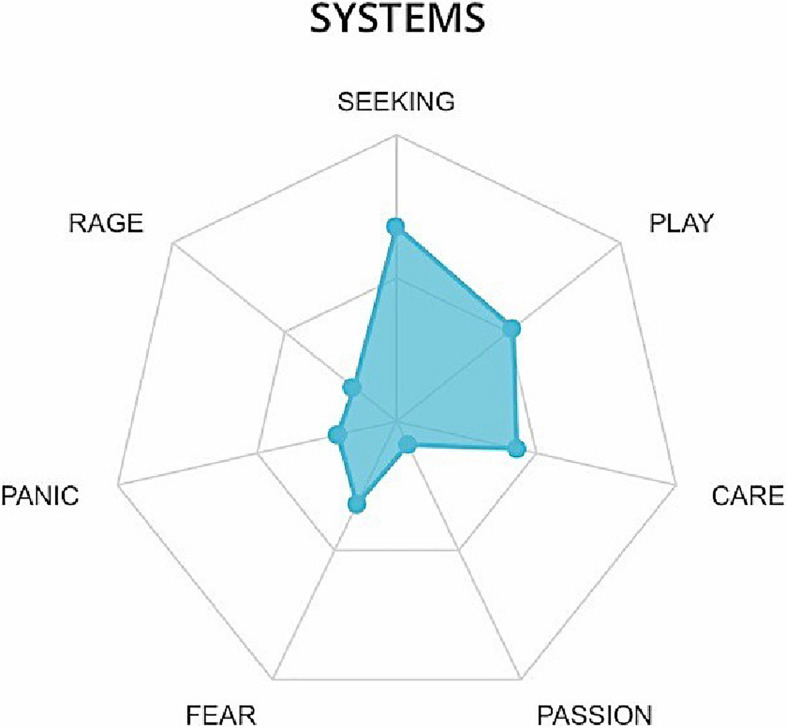
Cumulative web graph relating to all participants.

A cumulative web graph (Figure 4) is used to gather data relating to all participants’ emotional systems activation. The web graph is derived from the correspondence between chosen emotions and emotional systems as shown in Table 1.

#### Team Analysis: Tags and Affective Systems

We collected and analyzed recordings of four teams, that we named group A (GA), group B (GB), group C (GC), and group D (GD). The analysis is based on a qualitative analysis of group’s systems activation during time. Tag frequencies and relations with chosen emotions were also considered for the analysis.

**FIGURE 5:**
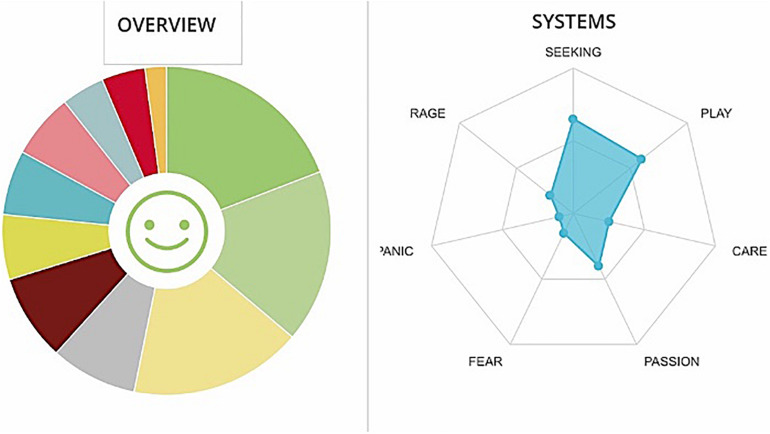
Graphs GA. The cumulative percentages of choice of emotions made by a team are represented in the pie chart (Different colors match to the different emotions as they are displayed in Figure 1) while the web graph shows the correspondence between chosen emotions and emotional systems.

**FIGURE 6:**
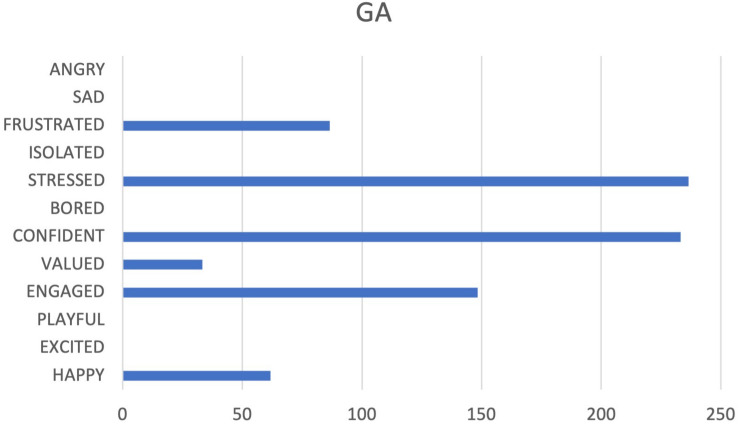
Cumulative frequencies of GA data input.

People on GA team seem to love their roles and appear to be happy when they work yet experiencing strong and mixed emotions (see Figures 5, 6). In fact, two opposite emotions are highlighted: feeling engaged and confident vs. feeling stressed. Workers report a good investment in their work, they respond positively when: they are given autonomy/are offered training/are offered new activities. However, from the correlation between tags and reported emotional states, it emerges that an information deficit is experienced as very negative. Tag’s analysis also revealed that an information deficit implicitly conveys a message of non-recognition to the participants. We hypothesized that the message of non-recognition can be connected to the activation of FEAR system.

The participants also seem to feel a strong sense of “pressure.” It was difficult to determine whether this can be traced back to characteristics of the job itself (possibly tight deadlines), or rather to characteristics of leadership (possibly lack of clarity of purpose).

**FIGURE 7:**
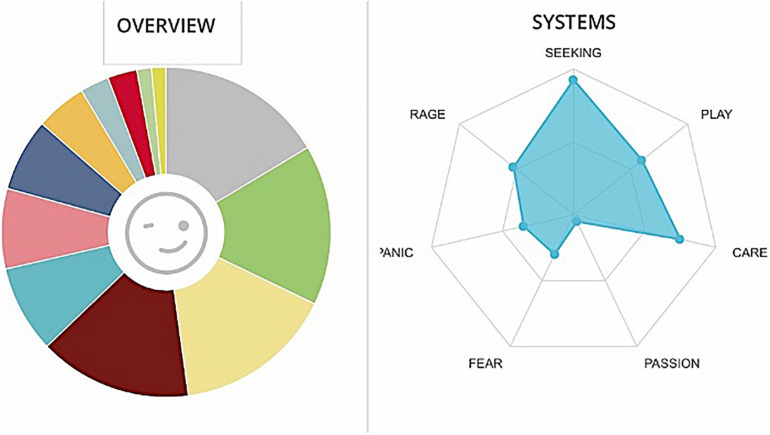
Graphs GB. The cumulative percentages of choice of emotions made by a team are represented in the pie chart (Different colors match to the different emotions as they are displayed in Figure 1) while the web graph shows the correspondence between chosen emotions and emotional systems.

**FIGURE 8:**
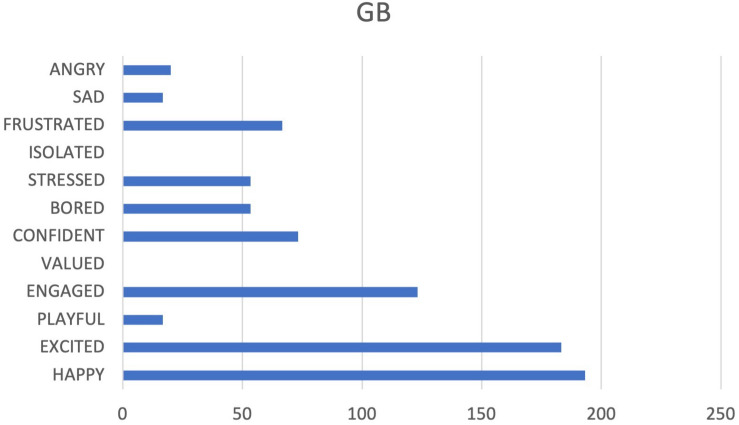
Cumulative frequencies of GB data input.

GB team appear to be very competitive, with a very strong investment in their job, but tied to a strong desire for external recognition (see Figures 7, 8). There could also be a very high cohesion, but it started from a perception of identity built on differences with respect to others: if a valorization of the group specificities could be positive, negative is this connotation of comparison/superiority with respect to other groups: this may lead to the creation of a strong team spirit on a team level, but a lower cohesion when it comes to the company as a whole. It is no coincidence that it is this team that expresses at the same time PASSION and RAGE. The commitment must be recognized as positive, but negative emotions must be monitored, since they are tolerable over a short period, due to contingent factors, but otherwise, it would be desirable if they were dealt with, trying to increase the system of CARE.

**FIGURE 9:**
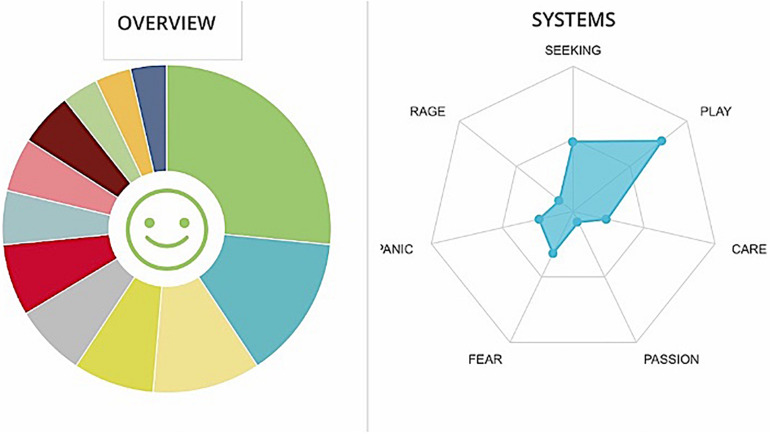
Graphs GC. The cumulative percentages of choice of emotions made by a team are represented in the pie chart (Different colors match to the different emotions as they are displayed in Figure 1) while the web graph shows the correspondence between chosen emotions and emotional systems.

**FIGURE 10:**
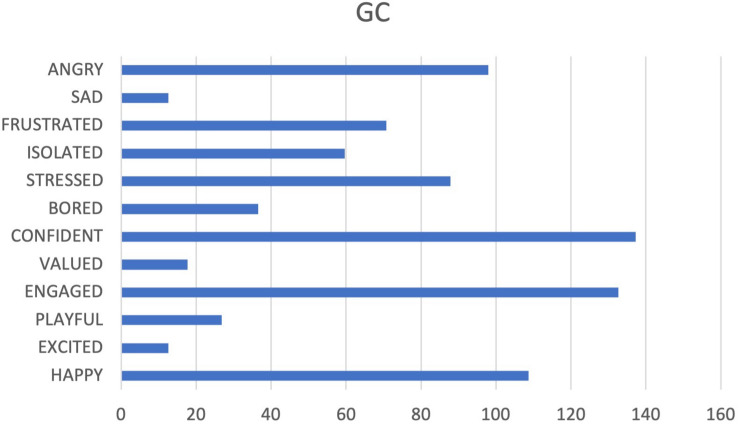
Cumulative frequencies of GC data input.

People belonging to GC team seem to have a good investment in their job and a focus on the task (see Figures 9, 10). They are not afraid of fatigue and work. Negative elements, such as anger and frustration, are present and are discernible not only from the declared lack of correct directives and adequate tools, but also in a sort of disconnection with any direction from leaders. Furthermore, the team also perceives its manager/headquarters as incompetent.

A sense of isolation from the organizational headquarters is perceivable. There is neither a competitive dimension nor a persecutory dimension, but a constant focus on work and conditions which promote organizational development.

**FIGURE 11:**
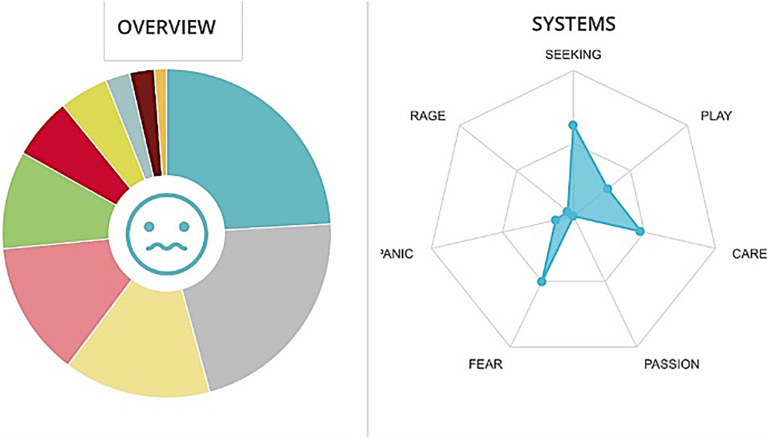
Graphs GD. The cumulative percentages of choice of emotions made by a team are represented in the pie chart (Different colors match to the different emotions as they are displayed in Figure 1) while the web graph shows the correspondence between chosen emotions and emotional systems.

**FIGURE 12:**
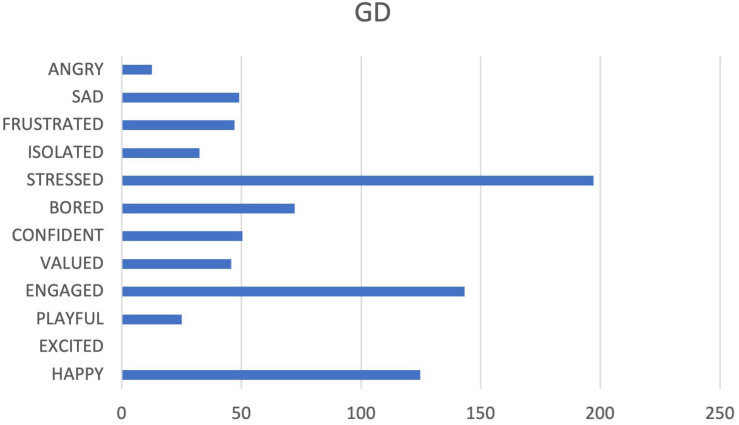
Cumulative frequencies of GD data input.

GD team has been very active in recording emotions. Positive emotions prevail, in particular the PLAY system and SEEKING system are active (see Figures 11, 12). We report the presence of stress, testified by the high level of the FEAR system and PANIC system.

### Discussion

#### Interpretation of Affective Systems Data

The cumulative company’s recordings show a high activation of the emotional systems of SEEKING, PLAY, and CARE, but also FEAR (cf., Figure 4). Following integration of the data of the chosen emotions and the associated tags, we propose the following considerations.

The SEEKING system appears to be well represented, yet in this system emotions with both positive (engaged) and negative (frustrated and bored) connotations are included. When analyzing in detail the recordings with integration of the tags, it emerges that recorded entries are equally divided between the positive and negative dimensions. Therefore, it appears to us that a good level of engagement and involvement is maintained for the achievement of objectives and the resolution of problems.

It should be noted that the activation of PLAY system – important for team functioning – is often linked to aspects external to work. If, on the one hand, it is inevitable that non-working aspects will affect people’s emotions, on the other hand it suggests that these emotions are not activated on the workplace. The activation of the PLAY system is instead of great importance, since it helps building group cohesion, i.e., a harmonized “teamwork.” Therefore, we could also assume that family and/or friendship dimensions (also) have a compensatory function with respect to work.

Although PLAY and SEEKING systems help to address problems in the “here and now” and help individuals and teams “invent” new solutions, it should be noted that these systems do not promote a rational and lasting solution to problems. Therefore, with respect to long-term business efficiency open questions, it would be useful, for instance, to strengthen CARE system to promote greater stability over time. It has also been consistently observed that routine activities elicit the CARE system. Even if the task is customary and demanding, whenever there is some clearly specified, predictable, and regular task in the organization, there is a greater sense of trust (e.g., a safe environment, a feeling of being able to face working from home).

It was also noted that the FEAR system was activated. We highlight that the activation is in relation to quite trivial tags, e.g., traffic. This suggests that the employees don’t feel to be safe enough. We think it would be useful to reinforce the CARE and PLAY systems, which could support a sense of belonging to a team, and therefore the belief that a team member can count on his/her colleagues.

The lack of a significant investment in the PASSION system is highlighted: this system is useful in supporting and encouraging the expression of creative solutions. These data would reinforce the hypothesis of an insufficient feeling of basic trust permeating the organization, since creativity and passion presuppose a condition of trust and reduced stress. In fact, in a condition of uncertainty, planning ability is usually suspended.

Except for a partial feedback when returning the data to all the participants, there were no means to confirm the validity of our data through comparison. For this reason, we asked to examine the documentation related to previous interventions and evaluations on the company (years 2016 and 2017). Of course, this was not deemed to be satisfactory, but highly conflicting data would have led us to question ourselves about our survey. We are well aware that over time significant changes may occur, but at the same time we know that problems are unlikely to be solved without an intervention. From past relations, a positive reference frame emerged, nonetheless criticalities have been pointed out in relation to organizational and functional aspects, in particular in the fields of communication, motivation, and evaluation. Therefore, the data we collected in a short time and with little participation from the company seem to be in line with the outcomes of more intensive studies, in addition to providing details about the single teams involved, as well as about the overall company.

#### Recommendations for the Company

The data and our interpretation were sent to all the subjects involved, and after a couple of months from the survey a feedback vis à vis was possible with some managers and the GB group, which was more directly involved for the specific job and affinity of the topics being researched.

Our recommendations for the company, which were communicated to the leadership, were as follows.

In a general, based on data collected in a transversal manner, with varying weights in the various teamwork situations, CARE and PLAY systems should be strengthened as a priority. These systems strengthen the sense of belonging, and of team cohesion, promoting a healthy expression of competitiveness within working groups. Initially, we suggested focusing attention on the management of communication between teams and line managers, and on interventions aimed at building or consolidating a sense of belonging. Communication should be treated with attention to feedback, in order to decline it in terms that respect the needs of the various working teams. This would also support the sense of belonging, through sharing the corporate mission and facilitating dialogue on the contribution of individual workers and teams in achieving corporate objectives.

During the feedback meeting with the company, the analysis of the general emotional states has been confirmed and found explanation in the company narrative of the events occurred during the data collection. In fact, in that period senior management underwent a revaluation of the executive systems, partly reinstating the former management system, and partly changing other sections. This took place without the staff having received precise information, or regular updates. Observing the data, a sense of “stalemate” is palpable, and is reflected by the reduced investment in LUST (PASSION). Moreover, external compensation (PLAY), presence of SEEKING in both positive and negative aspects and reassuring perception (CARE) of routine activities find, in the light of this information, a coherent interpretation as well as the proposal for a greater investment in communication, with the specific objective of strengthening the bond and the sense of belonging in employees.

During the face-to-face meeting with the managers and the GB group, the most significant achievement was that the participants recognized themselves in the descriptions, together with a certain surprise that our tool was able to return such a precise and articulated picture of the company’s functioning in a short period of time.

## Discussion

The use of basic emotional systems in the group and working dimensions had never been tested before, even if Panksepp himself foresaw this possible application. In fact, he wrote: “Certain emotional types seem to work best in specific roles and environments. Every manager needs to win the trust and respect of employees. Employees should feel that managers will help them with their problems at work, and managers should be confident that employees will meet their responsibilities. This implicit social contract is built on the mutuality of the CARE system. They must give each other what they need to feel secure and to excel. Managers also know the importance of team cohesion. Team days can support this process by fostering a spirit of PLAY, whereby members of a large working group share the opportunity to interact in more intimate and relaxed environments. This kind of playful interaction cements social bonds that are important for the solidarity of the workforce” (Panksepp and Biven, 2012 p. XX-XXI). Hoffman (2018) and Osnes (2020) applied affective neurosciences framework to improve the understanding of organizations and management dynamics. Their papers are mainly concerned with the study of leaders’ skills promotion and the analysis of emotion’s succession in leadership changes.

Strategies, behaviors, and attitudes that a leader can bring about in connection with the prevailing activation of some basic affective systems within the group are not very different from those identified by the literature as elements of a good leadership. The importance of leader behavioral integrity and its modulation with co-worker support and the level of perceived job autonomy (Choi et al., 2020), the effectiveness of a positive leadership, responding to the needs of competence, interrelation, autonomy and significance of the workers (Rahmadani et al., 2020.), the improvement of a supportive work climate in order to improve the workers well-being and organizations’ success (Lecca et al., 2020) are shareable evidences, which are also coherent with the setting we are proposing.

In our opinion, there are two aspects which change. The first one is the connection with the theoretical frame that attributes aspects and characteristics to the activation of primary emotional systems. Such a reference, like all models, suggests an interpretation of data instead of their plain description. A second difference depends on the specificity of the context: that is to say, the characteristics of an effective leadership are not set *a priori*, they are instead defined starting from a real, specific situation, at a well-defined time. This means that depending on the group functioning, the leader will find out which affective states are to be enhanced and which ones are to be limited and based on such analysis he/she will delineate his/her strategy. Therefore, in certain situations it might be more useful to activate the SEEKING system – proposing new challenges and valuing creativity and autonomy of workers – while in others it might be needed a greater activation of the CARE system –following the workers more closely, maybe using more defined and shared protocols and procedures.

The web app provides significant and timely information that allows the manager/leader to grasp some key elements related to business operation, thus identifying possible critical issues and hypothesizing lines of intervention. There would be increased confidence in management decisions about hypothesized lines of intervention if managers are informed by the tool about the affective activation systems.

In addition, the web app has the advantage of being capable of representing the evolution of affective dynamics over time. This feature is therefore suitable for present research developments, since an increasing approach is that of introducing time dimension in the study of affects and team dynamics. Shuffler et al. (2020) point out that “The time has arrived for a serious treatment of time in all aspects of research on teams” and that this new dynamic-focused approach to teams allows for a more nuanced understanding of how affect changes over time, how it influences and is influenced by other team phenomena over time, and how the effect of/on team members is shared. In particular, looking at the opportunity to picture the evolution of processes in team dynamics, new tools are being implemented, in addition to traditional methodological tools (i.e., surveys, interviews, case studies, and focus groups) like coding interaction and communication processes (Kolbe and Boos, 2019), simulation-based studies multi-method measurements, computational modeling, sociometric badges, computer-aided text analysis, and quantitative electroencephalography (Delice et al., 2019) analytical strategies like the SSG technique (Meinecke et al., 2019) recurrence analysis (Knight et al., 2016) and sequence analysis (Klonek et al., 2016). Generally speaking, these are instruments where the complexity of data collected requires an intensive work. The tool we propose provides an overview that might be non-exhaustive, but it is characterized by an easy data collection.

The tool provides analytical data for various teams over time, thus allowing tailor-made interventions. Although the data from our research relates to a small group of participants, we found a remarkable variety in the modes of functioning that characterize the different teams. Furthermore, the app does not only detect malfunctioning aspects, but also allows detecting the strengths of the systems analyzed. To plan and manage changes, it is necessary to be able to map reality, and to develop a sense of both fragility and resources.

It is advisable, in our opinion, to monitor and support motivation of participants, especially in case of surveys that last for a longer time. In fact, there has been a decline in the last 2 weeks of the survey, compared to the previous two, and specifically a dozen people have stopped registering their states. It should also be considered that the setting was that of a research, not an intervention required because of a problematic situation, which of course might have influenced the degree of motivation.

## Conclusion

The present work has important limitations – mainly because of the low sample size. Further research on different working situations is also necessary. However, from this pilot study, it became clear that with a small-time investment (less than a minute is enough to choose a state of affective activation on the app), anyone can register their emotions without the need to leave the current meeting/work environment. This ease of use is particularly significant for remote working, or during difficult periods such as the present pandemic emergency, when monitoring the health and efficiency of working organizations is particularly crucial, and when more traditional and face-to-face evaluation systems are not possible.

A limit of this study is that our agreement with the company did not allow for a longer monitoring period neither for the possibility to record data when the company was considering the introduction of some changes (at least on top management level). Nevertheless, we believe that the app could be useful to record changing in the dynamics over time, thanks to the differences in activation and configuration of the different basic affective states.

The tool ease of use, the possibility to perceive different affective activations in different groups, the possibility of recording in real time and monitoring over time seem to be interesting factors for a deeper study and for the application of this tool to other samples over a longer time period.

Finally, a risk of this instrument is that it can be thought of as a piano keyboard of affective states whose keys can be pressed to play the chosen melody. For example, to support the CARE system, a manager can organize a space for coffee breaks or schedule periodic meetings, in person or remotely, with staff. These actions can be truly supported by an intent to take care of employees, but these can also be merely instrumental actions taken to suppress in advance any request or proposal from workers. We believe it is unethical to lead a team and work on the emotions of others without being authentic and being involved in the relationships: it would be a lost opportunity. But authenticity can’t be prescribed.

In short, we think that in an organization we can “hear” and learn a great deal if we are able to participate in atmosphere and feelings, and not just paying attention to technical and financial aspects. Awareness of emotional flow opens up the possibility for a deeper dynamic understanding of teams’ functioning. We think that this level of understanding can support the development of more tailored strategies and employees’ health.

## Data Availability Statement

The raw data supporting the conclusions of this article will be made available by the authors, without undue reservation.

## Ethics Statement

Ethical review and approval was not required for the study on human participants in accordance with the local legislation and institutional requirements. The patients/participants provided their written informed consent to participate in this study.

## Author Contributions

Both authors listed have made a substantial, direct and intellectual contribution to the work, and approved it for publication.

## Conflict of Interest

The authors declare that the research was conducted in the absence of any commercial or financial relationships that could be construed as a potential conflict of interest.

## Footnotes

1 Capitalization is used to designate the various emotional systems to highlight the fact that these are instantiated as distinct neural entities rather than simple psychological concepts.
